# Non-Motor Manifestations in Idiopathic Dystonia with Focal Onset – A Pilot Study

**DOI:** 10.25122/jml-2020-0094

**Published:** 2020

**Authors:** Ovidiu-Lucian Bajenaru, Iulia Popescu-Olaru, Laura Dumitrescu, Elena Serban, Liviu Cozma, Florina Raicu, Relu Cocos, Ovidiu Bogdan Popescu

**Affiliations:** 1. Department of Clinical Neurosciences, “Carol Davila” University of Medicine and Pharmacy, Bucharest, Romania; 2. “Ana Aslan” National Institute of Geriatrics and Gerontology, Bucharest, Romania; 3. Department of Neurology, clinical Hospital Colentina, Bucharest, Romania; 4. Department of Medical Genetics, “Carol Davila” University of Medicine and Pharmacy, Bucharest, Romania; 5. Laboratory of Molecular Biology, “Victor Babes” National Institute of Pathology, Bucharest, Romania

**Keywords:** Dystonia, depression, cognitive impairment, quality of life

## Abstract

Recent studies emphasize an increased prevalence of non-motor symptoms in idiopathic dystonia with focal onset (IDFO), but their pathophysiological relationship is not clear.

We aimed to identify the prevalence of depression and neurocognitive impairment in a group of patients with idiopathic dystonia with focal onset and their impact on the patients’ quality of life.

This study represents a component of an ongoing research project – GENDYS. From the database of this project, we selected 48 patients 56.62+/-14.16 years old who have been examined clinically and using specific scales: Patient Health Questionnaire-9 (for depression), Montreal Cognitive Assessment - MoCA (for cognitive impairment), and a 5-degree analog scale for subjective perception of the severity of the disease. We conducted a descriptive cross-sectional study on patients with depression and cognition evaluated by the above-mentioned scales. We also performed a nested case-control analysis on 20 IDFO patients with and without at least moderate depression matched for age and gender; the cut-offs for depression were PHQ-9 score ≥10 and PHQ9 <5, for the depression group and the control group, respectively. The cut-off for MoCA was 26 points. 22 IDFO patients (46%) had depression; 54.5% of IDFO patients with depression had cognitive impairment, indicating a slight trend of increased cognitive impairment in those with depression compared to those without; the perception of the severity of disease was the greatest in patients with depression.

Depression is more prevalent in patients with IDFO and is associated with a worse perception of the disease severity.

## Introduction

Idiopathic dystonia with focal onset (IDFO) is defined as a heterogeneous group of movement disorders characterized by intermittent or sustained involuntary movements that determine abnormal dystonic postures and whose onset is focal in a muscle group. During evolution, these abnormal movements may keep the initial localization or extend to other muscles, which may define other topographic forms of dystonia (segmental, multifocal or generalized). Though most of the clinical studies have concentrated their attention on the motor abnormalities defining dystonia, during the last decade, more reports emphasizing the importance in many cases of associated nonmotor clinical manifestations have been published [1-10]; most of these symptoms were related to mood and cognition, but also olfactory, sleep disorders, fatigue, and other sensory symptoms. Their etiopathogenic significance and importance on the patients’ quality of life are not yet completely understood.

We do not have enough data to understand if these non-motor manifestations are psychological consequences of dystonia or intrinsic phenomena determined by the pathologic changes leading to both motor and non-motor symptoms, or if both mechanisms are valid [11]. The clinical reality indicates that these complex clinical manifestations are important not only for the pathophysiologic relationships but also to identify in clinical practice the factors able to influence the quality of life and which can interfere with the social stigma feeling, often noted in these patients in other studies [12-17]. In particular, in one of the cited studies [17], these aspects have reconfirmed using the International Classification of Functioning, Disability and Health (ICF) and emphasized that patients complained not only of motor dysfunction but also a series of “mental dysfunctions” in daily activities, subjectively perceived as being worsened not mainly by dystonia but by the disturbances in their interpersonal relations and interactions.

In our study, we aimed to analyze if in a group of Romanian patients with IDFO, there is a significant presence of some particular mood and neurocognitive symptoms and if their presence could be useful to identify some particular clinical phenotypes of dystonia.

## Material and Methods

This study represents a component of an ongoing larger research project (PN-III-P4-ID-PCE2016-0696), identified as GENDYS. This project began in 2016, after obtaining approval from the Ethics Committee of the Clinical Hospital Colentina Bucharest; the selected patients have been enrolled only after signing informed consent. Their selection was consecutive among all patients diagnosed with IDFO using The International Parkinson and Movement Disorder Society (MDS) criteria [18, 19], until February 28, 2019. The total number of subjects enrolled in the GENDYS project when we began the analysis for the actual study was 116. The patients were subjected to a general and neurologic examination, and some specific clinical scales internationally validated that have been used for non-motor symptoms, including the Patient Health Questionnaire-9 (PHQ-9) for depression and Montreal Cognitive Assessment (MoCA) for neurocognitive disorders. All these data have been included in a large database for the GENDYS project. For the analysis of the data necessary to the actual study, we selected from this database 48 patients - 11 males (M) and 37 females (F) with an average age of 56.62 +/- 14.16 years old, who have been analyzed in a descriptive cross-sectional study; the selection of these patients was based on the availability of above-mentioned clinical scales for depression and cognition. In the next step, we performed a nested case-control analysis in 20 patients with IDFO, with and without depression, at least moderate (PHQ-9 score ≥10), matched for age and gender, which have been selected from a group of 22 patients with available values for this score (2 patients have been eliminated in order to match the two comparable groups for statistical analysis). To define the depression group, we considered a cut-off value for PHQ-9 of at least 10 points (meaning moderate, moderate-severe, and severe depression) and less than 5 points for the control group (without depression). To evaluate the subjective perception of the dystonia severity, we used a 5-degree analog scale (from 0 to 4), values higher than 2 indicating an increased subjective severity, and these values have been correlated with the scores for depression. For the statistical analysis of these data, we used the chi-square test due to the small number of subjects available.

## Results

In the cross-sectional analysis, we obtained the following distribution of the IDFO subtypes: 20 patients (4 M and 16 F) with cervical dystonia, 23 patients (3 M and 20 F) with craniofacial dystonia and 5 patients (3 M and 2 F) with upper limb dystonia. According to the values of the PHQ-9 scale, 22 patients (i.e., 46% out of the 48 patients) also had depression (PHQ-9 score ≥5), more prevalent in females: 18 F vs. 4 M (49% vs. 36%, p=0.06) (Table 1).

**Table 1:** Prevalence of depression (PHQ-9 score).

**Patients**	**With depression (PHQ-9** ≥ **5)**			**Without depression (PHQ-9 < 5)**		
**M**	4 patients	36%	22 pts.	7 patients	64%	26 pts.
**F**	18 patients	49%		19 patients	51%	

The 20 patients selected for the nested case-control analysis (i.e., 2 M and 8 F) per group had an average age of 51.40 +/- 14.37 years old for the study group and 50.90 +/- 12.88 years old in the control group.

The analysis of the neurocognitive performance evaluated by the MoCA score has shown a neurocognitive impairment (score <26 p) in almost half of these patients with a slightly increased prevalence in those younger than 65 years old (57% vs. 43%, p=0,1). The correlative analysis between the neurocognitive impairment and the presence of depression did not show significant differences between cognitive impairment associated with depression (54.5%) compared to dystonia without depression (45.5%) (Table 2).

**Table 2:** Cognitive status (MoCA score; cut-off: <26).

**Patients with depression (PHQ-9** ≥ **5)**			**Patients without depression (PHQ-9 < 5)**			**Total**	
12 patients		54.5%	10 patients		45.5%	22 patients	100%
Age	Score		Age	Score			
≥65 years	4 patients	50%	≥65 years	4 patients	50%	8 patients	100%
<65 years	8 patients	57%	<65 years	6 patients	43%	14 patients	100%

In the nested case-control study, we included only patients with significant depression (Table 2), and the values of the MoCA score indicating a significant cognitive impairment (<26 p) were present in almost half of the patients (9 subjects); comparing the subjects with depression to those without depression, we have noticed a slight increase of the cognitive impairment level in those with depression (50% vs. 40%, OR=1.5; p=0.65; 95% CI= 0.25-8.82). Patients with craniofacial IDFO had a non-statistical significant increased prevalence of depression than patients with cervical dystonia (60% vs 30%, OR=3.5; p=0.18; 95% CI= 0.55-22.30) (Table 3). A slight increase in depression was present in patients with facial IDFO compared to its prevalence in all IDFO cases. In Table 4, we present the comparative data related to the patients’ subjective evaluation of the severity of depression using a 5-degree analog scale; we notice that this perception has the greatest severity in patients with depression compared to those without depression.

**Table 3:** Distribution of types of IDFO with and without depression.

**Patients with at least moderate depression (PHQ-9** ≥ **10)**				**Patients without depression (PHQ-9 < 5)**			
M	2	Cervical dystonia	2	M	2	Cervical dystonia	1
		Craniofacial dystonia	0			Craniofacial dystonia	0
		Upper limb dystonia	0			Upper limb dystonia	1
F	8	Cervical dystonia	1	F	8	Cervical dystonia	5
		Craniofacial dystonia	6			Craniofacial dystonia	3
		Upper limb dystonia	1			Upper limb dystonia	0

**Table 4:** Severity of perception.

**Patients with at least moderate depression (PHQ-9** ≥ **10)**		**Patients without depression (PHQ-9 < 5)**	
average score ± S.D.	3.4 ± 0.66	average score ± S.D.	2.5 ± 0.5
increased perception	9	increased perception	5
(score = 3-4)		(score = 3-4)	
moderate perception	1	moderate perception	5
(score = 1-2)		(score = 1-2)	

## Discussions

In this pilot study that investigated patients with IDFO, we noticed the presence of mood disorders (especially depression) and mild neurocognitive impairment in almost half of the studied group. As long as the number of patients available for this study is relatively small for robust statistical analysis, we cannot have clear-cut conclusions, but a remarkable trend related to the presence of depression and mild cognitive impairment is evident. These data are in accordance with the conclusions of most of the other studies we have cited in this paper, published in the last years. In some of these studies [8, 17, 20], the authors noticed that in most of the patients who affirmed significant restrictions in their daily life activities, these are not due to dystonia by itself, but to the alteration of their social and interpersonal interactions that are not determined by motor symptoms; some of these patients considered these dysfunctionalities to be severe. In other studies [21-23], the presence of cognitive dysfunctions was considered to be most often significant, but without elucidating their cause. In one of these studies [22], the authors also cite previous reports suggesting that decreased cognitive performance could be secondary to the psychological impact of motor and/or mood disorders associated with dystonia. Other authors [24-28] have shown that psychiatric and other non-motor comorbidities have an increased prevalence and are a predictor for the quality of life in particular in cervical dystonia, even more than the severity of dystonia. The observations of our study are consistent with these conclusions. Moreover, in our study, we emphasize the observation that the subjective perception of the disease severity seems to be greater in patients with IDFO, in particular with craniofacial dystonia. In one of the above-cited studies [24], it has been noticed that the prevalence of psychiatric symptoms is significantly higher (64% vs. 28%, p=0.001) in patients with dystonia compared to those without dystonia, depression and anxiety being the most constant. These authors also noticed that the most important predictors of decreased quality of life have been the severity of depression, along with the severity of pain and disability, but not the severity of motor symptoms. Other authors added that not only these factors negatively influence the quality of life, but also other non-motor symptoms such as social phobia problems and sleep disorders, which are often not diagnosed in patients with dystonia [29-32].

Some of the results of this study were presented as a poster presentation at the MDS Congress in 2019 [33].

## Conclusions

Our study, though limited by the small number of patients available for the statistical analysis, shows an important trend of the presence of mood disorders - in particular depression, and mild neurocognitive impairment as associated non-motor symptoms in patients with IDFO and this conclusion is in accordance with data published in many recent studies. Moreover, in our study, we have shown that the presence of depression (moderate, moderate-severe or severe) associated with IDFO has a negative impact in the subjective perception of the severity of dystonia and the quality of life.

The presence of mild cognitive impairment seems to be another clinical feature in patients with IDFO, even if their intensity, at least that observed in our study, is not increased.

The potential answer to the question if the presence of these non-motor symptoms is an intrinsic feature of at least some IDFO phenotypes could be clarified by more profound genetic studies using Next Generation Sequencing (NGS) which, at least hypothetically, could identify genes whose pathological changes could better correlate to distinct IDFO phenotypes with or without non-motor symptoms [34]. In fact, this is one of the final targets of the GENDYS project [35, 36]. This idea is also supported by preliminary data from other recent studies [37-39], which suggest the existence of correlations between genetic abnormalities and the presence of some psychiatric symptoms, especially depression, in some forms of IDFO, also correlated with abnormalities in the serotoninergic and dopaminergic systems.

## Acknowledgments

This study represents a component of GENDYS, which is an ongoing research project (PN-IIIP4-ID-PCE-2016-0696) funded by the Executive Unit for Financing Higher Education, Research, Development and Innovation (UEFISCDI), Romania.

## Conflict of Interest

The authors declare that there is no conflict of interest.
